# Bacterial Community Structure in Soils With Fire‐Deposited Charcoal Under Rotational Shifting Cultivation of Upland Rice in Northern Thailand

**DOI:** 10.1002/ece3.70851

**Published:** 2025-02-05

**Authors:** Noppol Arunrat, Toungporn Uttarotai, Praeploy Kongsurakan, Sukanya Sereenonchai, Ryusuke Hatano

**Affiliations:** ^1^ Faculty of Environment and Resource Studies Mahidol University Nakhon Pathom Thailand; ^2^ Department of Highland Agriculture and Natural Resources, Faculty of Agriculture Chiang Mai University Chiang Mai Thailand; ^3^ Laboratory of Terrestrial Ecosystem Modeling, Research Faculty of Agriculture Hokkaido University Sapporo Japan; ^4^ Laboratory of Soil Science, Research Faculty of Agriculture Hokkaido University Sapporo Japan

**Keywords:** bacterial community, charcoal, fire, rotational shifting cultivation

## Abstract

Rotational shifting cultivation (RSC) is a traditional agricultural practice in mountainous areas that uses fire to clear land after cutting vegetation for cultivation. However, few studies have assessed the effect of fire‐deposited charcoal on the diversity and composition of soil microbial communities, and none have been conducted in Thailand. Therefore, this study was conducted 1 year after a fire in an abandoned 12‐year RSC in Chiang Mai Province, northern Thailand. Charcoal samples were collected from the surface litter layer, while charcoal‐soil mixtures were taken from the surface soil (0–2 cm). Soil samples from 2 to 7 cm captured the charcoal‐soluble layer, and samples from 7 to 15 cm represented soil without charcoal incorporation. The results revealed that charcoal led to higher pH and electrical conductivity in the charcoal layer, with notable differences in soil texture across layers, including the highest sand and silt content in the charcoal‐mixed soil layer (0–2 cm). Soil organic matter and total nitrogen were significantly higher in the charcoal‐mixed layer compared to deeper layers, indicating improved nutrient retention due to charcoal presence. Enhanced microbial diversity was observed in the charcoal and charcoal‐mixed soil layers, with Proteobacteria, Chloroflexi, and Planctomycetota dominating across all soil samples. The bacterial genus *Ilumatobacter* exhibited significant changes in abundance in the charcoal layer. Additionally, *Pseudolabrys* was more abundant in charcoal‐leached soil, while JG30a‐KF‐32 showed greater abundance in soil without charcoal. Shifts in Proteobacteria and Planctomycetota abundance were evident in the charcoal leaching and non‐charcoal layers. Network analysis indicated more complex bacterial interactions in the charcoal‐mixed soil layer, with reduced network complexity observed in the charcoal leaching layer and the layer without charcoal. These findings imply that charcoal provides a favorable environment for diverse and interactive bacterial communities, potentially benefiting soil health and fertility recovery in RSC fields.

## Graphical abstract

Enhanced microbial diversity was observed in the charcoal and charcoal‐mixed soil layers, with Proteobacteria, Chloroflexi, and Planctomycetota dominating across all soil samples. Specific bacterial genera, such as *Pseudorhodoplanes* and *Ilumatobacter*, showed significant changes in abundance due to charcoal presence.

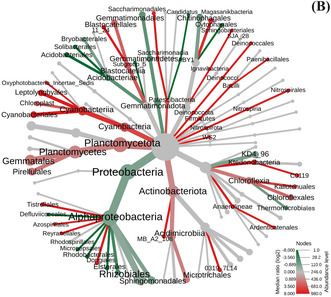

## 1 Introduction

Fire is an essential tool in rotational shifting cultivation (RSC), particularly in Northern Thailand, where it is used for periodic clearing and burning of forested areas to create arable land (Fukushima et al. 2008; Arunrat, Sereenonchai, and Hatano 2022; Arunrat et al. 2023a). This practice serves multiple purposes: controlling pests (Howard 1996), shaping vegetation patterns (Brussaard et al. 2007; Fukushima et al. 2008), releasing nutrients from organic matters (OMs) (Muqaddas et al. 2019; Roth et al. 2023; Strydom, Smit, and van Tol 2024), and stimulating the growth of specific plant species (Bárcenas‐Moreno et al. 2016; Garcia‐Pausas, Romanyà, and Casals 2022). Additionally, it alters soil physiochemical properties (Fox, Darboux, and Carrega 2007; Alcañiz et al. 2016).

Following the burning phase, a fallow period allows the land to recover its fertility through the addition of ash (Styger et al. 2007; Grogan, Lalnunmawia, and Tripathi 2012; Rietl and Jackson 2012). Ash produced from burning OM contains essential nutrients like nitrogen, carbon, phosphorus, and potassium, which are rapidly released into the soil, improving nutrient availability for plant uptake (Cade‐Menun et al. 2000; Muqaddas et al. 2019; Xu, Elberling, and Ambus 2022). However, nitrogen is typically not found in significant amounts in ash after burning, as it is released into the atmosphere during combustion as N₂ and NOx. This volatility contrasts with other minerals such as Ca, K, Mg, Na, and P, which are more likely to remain in the ash (Kalidas‐Singh et al. 2021). This immediate nutrient boost supports the initial stages of crop growth and helps in the rejuvenation of the soil ecosystem (Hamman, Burke, and Knapp 2008; Cutler et al. 2017). However, the process also results in the deposition of fire‐derived materials such as charcoal into the soil. Charcoal, a byproduct of incomplete combustion, is notable for its persistence in the soil over extended periods (Santín et al. 2015; Pyle et al. 2017; Faghih et al. 2019). Charcoal can influence the breakdown of unburnt OM (Wardle, Nilsson, and Zackrisson 2008) and impede the loss of native OM (Pluchon et al. 2016). Its porous structure and high surface area significantly influence ecological and soil characteristics (Wardle, Nilsson, and Zackrisson 2008; Makoto et al. 2011; Briggs, Breiner, and Graham 2012). The interaction between charcoal and soil microbes is particularly critical, as it can lead to substantial changes in soil microbial dynamics (Zackrisson, Nilsson, and Wardle 1996; Wardle, Zackrisson, and Nilsson 1998; Makoto et al. 2010; Serrani et al. 2023). The presence of charcoal in soil can alter its physical and chemical properties (Tryon 1948; MacKenzie et al. 2008), potentially affecting the composition and functionality of soil bacterial communities. These communities play a critical role in nutrient cycling (Pérez‐Valera et al. 2020), OM decomposition (Condron et al. 2010), and overall soil health. However, research on the interactions between charcoal and native OM under agricultural conditions remains limited, particularly in long‐fallow rotational cultivation systems, which are still understudied.

Various physical, chemical, and biological soil attributes serve as reliable indicators of changes in soil processes following natural disturbances, helping to measure the recovery of soil functions (Hubbert et al. 2005; Mataix‐Solera et al. 2011; Agbeshie et al. 2022; Roth et al. 2023). Fire‐induced alterations in soil quantity and quality significantly influence ecosystem responses post‐fire (Certini 2005; Jiménez‐Pinilla et al. 2016; Moya et al. 2021). However, a combination of physicochemical and microbiological indicators is crucial for a thorough understanding of soil recovery after fire (Mataix‐Solera et al. 2009; Goberna et al. 2012). Microbial indicators, such as soil microbial biomass, activity, and community composition, respond rapidly to disturbances, making them excellent markers for detecting environmental changes (Fioretto, Papa, and Pellegrino 2005; Aboim et al. 2008; Chiroma and Alhassan 2023).

In RSC systems with wider fallow periods, post‐fire ecosystem recovery involves complex microbial interactions (Grogan, Lalnunmawia, and Tripathi 2012; Saplalrinliana et al. 2016; Lungmuana et al. 2017). However, important questions remain unanswered: (1) which nutrients remain after one year following the fire in the RSC hillslope area? and (2) could the deposited charcoal induce higher complexity of bacterial networks at the surface compared to deeper layers?. The objective of this study was to investigate the bacterial community structure and composition in charcoal‐containing soil, by examining various layers: the surface soil layer, the charcoal‐soluble layer, and deeper layers of soil without charcoal. We hypothesized that: (H1) fire‐deposited charcoal could retain soil OM, soil availability, and exchangeable nutrients in the surface soil, and (H2) high‐complexity networks of soil bacteria would be detected in the charcoal‐mixed soil layers due to nutrient availability and pH regulation for bacteria. This study offers valuable insights into the impact of fire on RSC fields, with the aim of guiding sustainable land management practices and enhancing the resilience of RSC systems in Northern Thailand.

## 2 Materials and Methods

### 2.1 Study Areas and Site Selection

The study area is located in Ban Mae Pok, Ban Thab Subdistrict, Mae Chaem District, Chiang Mai Province, Northern Thailand. According to data from the Thai Meteorological Department in Chiang Mai Province (Doi Ang Khang and Mueang Chiang Mai stations) for 2021–2022, annual rainfall ranged from 1105 to 2688 mm, primarily occurring during the rainy season from May to October. During the winter season (October to February), minimum temperatures ranged from 3.2°C to 22.1°C, whereas in the summer season (February to April), maximum temperatures ranged from 35°C to 40°C (Arunrat et al. 2024).

**FIGURE 1:**
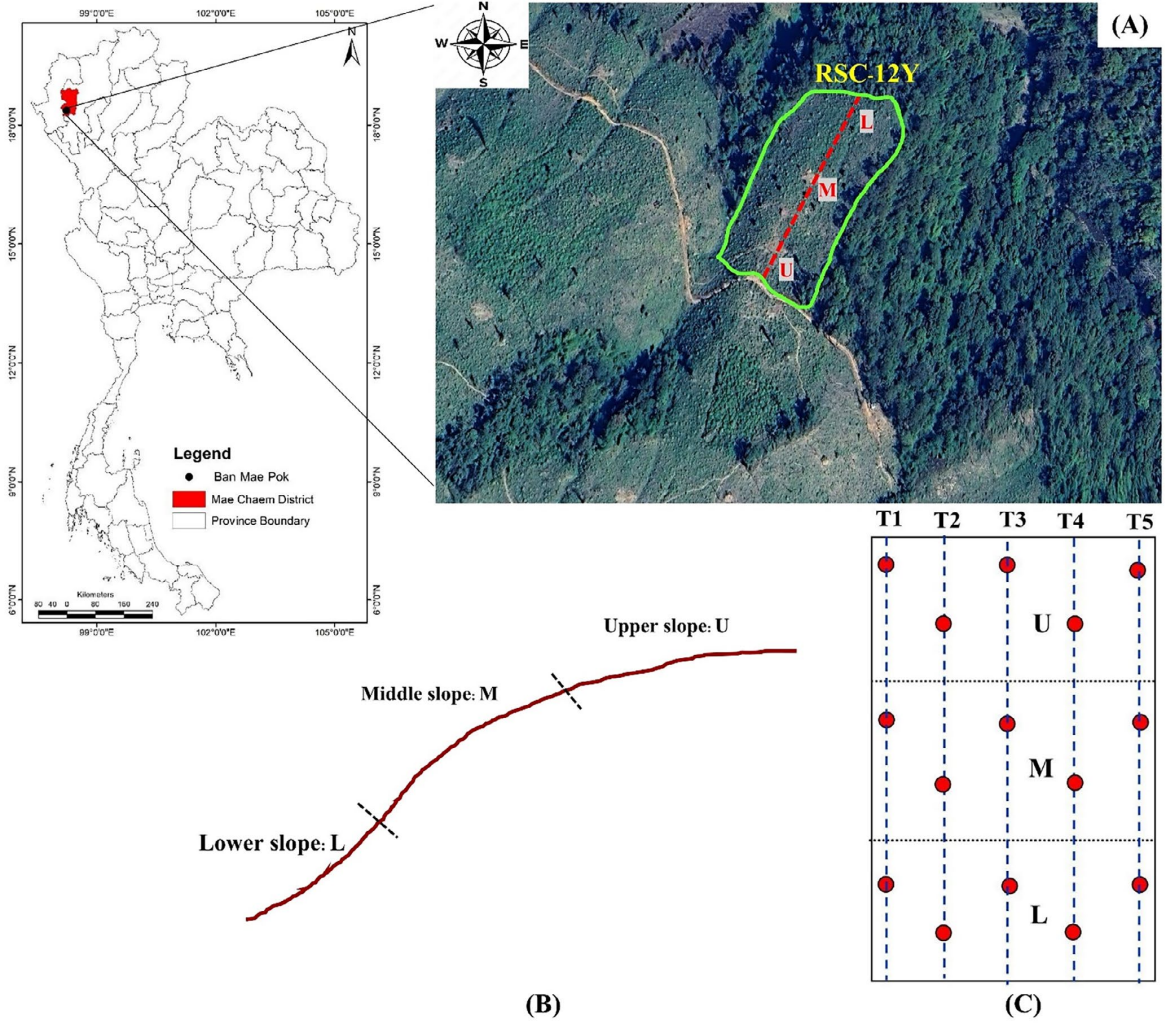
Study area. (A) location of RSC‐12Y, (B) three slope positions, (C) transects, and sampling points.

**FIGURE 2:**
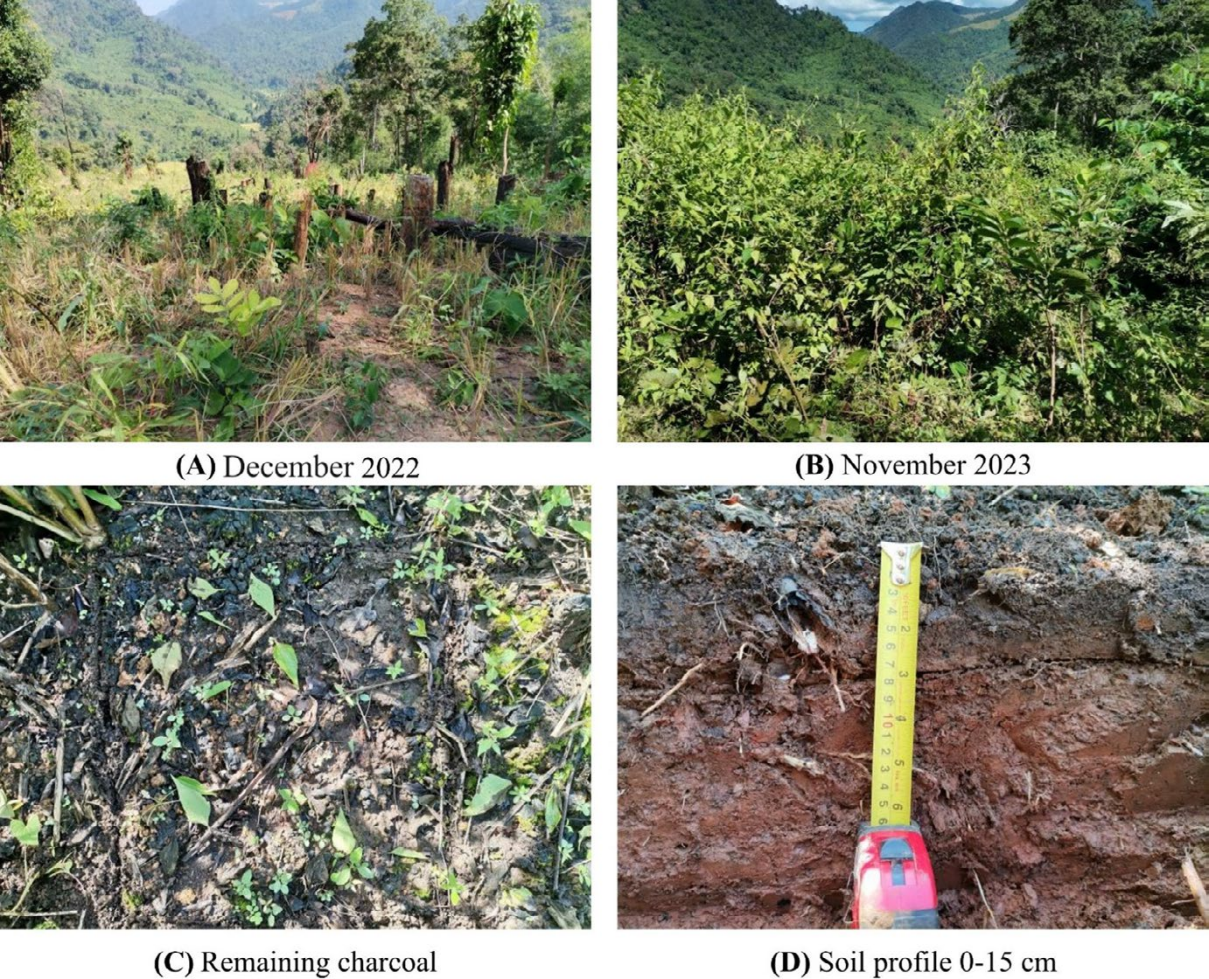
Recovery of RSC‐12Y, remaining charcoal, and soil profile for sampling.

Site RSC‐12Y (18°23′17″N, 98°11′41″ E; elevation 692 m a.s.l; slope gradient 28%) was used for this study (Figure 1A) and covers an area of 75 m × 170 m. This field has been left fallow for 12 years following the upland rice harvest. In 2022, it was cleared, burned, and cultivated for upland rice (dryland rice), with seeds randomly sown by hand. The rice crop was harvested in December 2022, after which the field was left fallow to allow for vegetation recovery again (Figure 2A) (Arunrat et al. 2023b).

### 2.2 Experimental Design, Remaining Charcoal, and Soil Sampling

The charcoal and soil sampling were conducted in November 2023, 1 year after the fire (Figure 2B). Five transects were established along the upper slope, middle slope, and lower slope of the RSC‐12Y field (Figure 1B,C) to minimize variation in soil nutrients caused by runoff from higher to lower elevations, which can impact the richness and diversity of soil bacteria along the slopes (Arunrat et al. 2023a). At each transect, three plots (1 m × 1 m) were marked at the upper slope, middle slope, and lower slope positions for sampling. From each plot at each slope position, samples were collected from four layers along the soil profile, each exhibiting varying amounts of charcoal. The vertical distribution of charcoal throughout the soil horizons was identified by examining specific physical features such as color and size of charcoal, which were then used for layer classification. Stones, grasses, roots, and the other residues were removed manually. Steel knives were used to collect the samples. At the surface litter layer, only the remaining charcoal samples were collected, without any soil or other residues (Figure 2C). At the surface soil layer (0–2 cm), samples of tiny charcoal particles mixed with soil surface were taken. At a depth of 2–7 cm, soil samples were collected to capture the accumulation of the charcoal‐soluble layer. At the bottom of the soil profile, soil samples were collected from a depth of 7–15 cm, representing soil without charcoal incorporation (Figure 2D). A sample from each layer at the upper slope, middle slope, and lower slope positions was combined to obtain one composite sample per transect. Approximately 1 kg of soil from each layer was collected in plastic bags for physical and chemical property analysis. Moreover, about 100 g of soil from each layer was stored in zip‐lock plastic bags and kept at −20°C for DNA extraction. A total of 20 samples were collected, with five replications for each layer.

### 2.3 Analysis of Soil Physicochemical Properties

The hydrometer method was employed for soil texture analysis. Soil pH was measured using a pH meter with a 1:1 soil‐to‐water ratio (National Soil Survey Center 1996). Saturation paste extracts and an EC meter were used to determine electrical conductivity (ECe) (Soil Survey Staff 2014). The micro‐Kjeldahl method was used to measure total nitrogen (TN) content (Soil Survey Staff 2014). Exchangeable calcium (exch. Ca), magnesium (exch. Mg), and potassium (exch. K) were analyzed through atomic absorption spectrometry (AAS) after extraction with 1 N NH_4_OAc at pH 7.0 (Jones Jr. 2001; Thomas 1982). Available phosphorus (avail. P) was measured by the molybdate blue method following Bray II extraction (Jones Jr. 2001; Bray and Kurtz 1945). Organic carbon (OC) was quantified using potassium dichromate (K_2_Cr_2_O_7_) in sulfuric acid (H₂SO₄) (Jones Jr. 2001; Walkley and Black 1934) and was then converted to OM by multiplying by a factor of 1.5 (Shamrikova et al. 2022).

### 2.4 DNA Extraction, Bacterial 16 s Amplification, Sequencing, and Bioinformatics Analysis

Extraction of soil DNA (0.5 g) was performed using the DNeasy PowerSoil Pro DNA Kit by Qiagen. The extracted DNA underwent amplification focusing on the V3‐V4 region of the 16S rRNA gene with the use of primers 341F (5′‐CCTAYGGGDBGCWSCAG) and 805R (5′‐GGACTACNVGGGTHTCTAAT‐3′), as outlined by Klindworth et al. (Klindworth et al. 2013). The amplification condition included an initial denaturation step of 2 min at 98°C, followed by 30 cycles of 98°C for 20 s, 60°C for 30 s, and 72°C for 60 s, followed by a single final extension step at 72°C for 1 min. Subsequently, 16S amplicons were purified using sparQ Puremag Beads (QuantaBio, USA) and indexed using 2.5 μL of each Nextera XT index primer in a 50 μL PCR reaction, followed by 8–10 cycles of PCR conditions above. The final PCR products were cleaned, pooled, and diluted to a final loading concentration of 4 PM. Cluster generation and 250‐bp paired‐end read sequencing were performed on an Illumina MiSeq at Omics Sciences and Bioinformatics Center (Chulalongkorn University, Bangkok, Thailand). The sequencing data relevant to this research are available at the National Center for Biotechnology Information (NCBI), recorded under the BioProject accession number PRJNA1085479.

The bacterial 16S rRNA gene sequences were analyzed using the QIIME2 software, version 2022.2 (Estaki et al. 2020). Initial preprocessing involved the removal of primer sequences using the Cutadapt tool (Martin 2011). Following this, sequences underwent quality control, assembly, and chimera detection using the DADA2 plugin (Callahan et al. 2016). Sequences with resemblance were classified into amplicon sequence variants (ASVs). Subsequently, ASVs comprising fewer than two sequences were excluded from the dataset. Bacterial classifications were assigned based on the Silva database version 138 (Quast et al. 2013). Normalization of ASV counts was performed to equalize depth across samples using the rarefy plugin.

### 2.5 Statistical Analysis

Statistical analyses were conducted using R program (R Development Core 2019), PAST (Hammer, Harper, and Ryan 2001), and STAMP software (Parks et al. 2014). A one‐way ANOVA followed by Tukey's HSD post hoc tests was conducted to assess significant differences in soil texture and chemical properties across different layers: charcoal, charcoal‐mixed soil, charcoal‐leached soil, and soil without charcoal. Alpha diversity indices, including observed richness and Shannon indices, were statistically compared among the treatments using ANOVA. The bacterial communities were analyzed and visualized by non‐metric multidimensional scaling (NMDS) based on the Bray–Curtis distance. The differences in compositions were tested using NPMANOVA, with the *p*‐value based on 9999 permutations.

The heat tree analysis utilizes the hierarchical nature of taxonomic classifications to quantitatively (employing median abundance) and statistically (applying the non‐parametric Wilcoxon Rank Sum test) illustrate the taxonomic differences between microbial communities (Foster, Sharpton, and Grünwald 2017). The STAMP software was employed to identify operational taxonomic units (OTUs) and specified genera that significantly changed in relative abundance due to the treatments. The analysis was conducted with default parameters. Redundancy analysis (RDA) was utilized to assess the impact of soil properties on the compositions of soil bacterial communities, with the significance of these correlations verified through the Mantel test.

### 2.6 Network Analysis of Bacterial Co‐Occurrence

To study the interactions within bacterial communities for each treatment, co‐occurrence network analysis was performed. Spearman's rank correlations were computed for bacterial ASVs identified within each treatment. Only correlations exhibiting a coefficient (σ) exceeding 0.7, alongside a significant *p*‐value (*p* < 0.05), were deemed strong and subsequently utilized to construct the co‐occurrence networks. Visualization of the network was achieved using Gephi (Bastian, Heymann, and Jacomy 2009), employing an undirected network model and the Frauchterman–Reingold layout. Within these networks, each node signifies a genus, and each edge (or link) denotes the association between genera.

## 3 Results

### 3.1 Soil Physiochemical Properties

The percentages of sand, silt, and clay in different soil layers affected by charcoal were presented in Table 1. For the charcoal layer, no data was measured. In the charcoal‐mixed soil layer (0–2 cm), the soil contained 20.6% sand, 46.6% silt, and 32.8% clay, with these values being significantly different from those in deeper layers. In the charcoal leaching layer (2–7 cm), the sand content decreased to 18.4%, silt content to 39.1%, and clay content increased to 42.5%, showing significant differences from the 0–2 cm layer but not from the 7–15 cm layer. In the layer without charcoal (7–15 cm), the sand content was 18.7%, silt content was 35.4%, and clay content was 45.9%, values similar to the 2–7 cm layer.

**TABLE 1:** Soil texture of each layer (mean ± standard deviation).

Properties	Charcoal	Charcoal‐mixed soil (0–2 cm)	Charcoal leaching (2–7 cm)	Without charcoal (7–15 cm)
Sand (%)	—	20.6 ± 0.7^a^	18.4 ± 1.9^b^	18.7 ± 0.8^b^
Silt (%)	—	46.6 ± 1.4^a^	39.1 ± 1.1^b^	35.4 ± 0.6^b^
Clay (%)	—	32.8 ± 1.3^b^	42.5 ± 2.5^a^	45.9 ± 1.1^a^

^a‐b^ Denotes significant differences (*p ≤* 0.05) determined by one‐way ANOVA followed by Tukey's HSD post hoc test.

The charcoal layer has a significantly higher pH (10.64) and ECe (5.95 dS/m) compared to the deeper layers. SOM content was significantly higher in the charcoal‐mixed soil layer (6.59%) than in the charcoal layer (5.34%). SOM decreased significantly in the charcoal leaching layer (2.98%) and further in the without charcoal layer (1.18%). TN followed a similar pattern, being significantly higher in the charcoal‐mixed soil layer (0.31%) compared to the charcoal layer (0.14%) and much lower in the charcoal leaching (0.11%) and without charcoal (0.06%) layers. Avail.P, Exch.K, Exch.Ca, and Exch.Mg were significantly highest in the charcoal‐mixed soil layer and declined with increased depth (Table 2).

**TABLE 2:** Soil chemical properties of each layer (mean ± standard deviation).

Properties	Charcoal	Charcoal‐mixed soil (0–2 cm)	Charcoal leaching (2–7 cm)	Without charcoal (7–15 cm)
pH (1:1)	10.64 ± 0.86^a^	6.16 ± 0.25^b^	5.17 ± 0.09^b^	4.60 ± 0.09^c^
ECe (dS m^−1^)	5.95 ± 0.27^a^	0.44 ± 0.06^b^	0.13 ± 0.02^c^	0.08 ± 0.02^d^
SOM (%)	5.34 ± 0.41^b^	6.59 ± 0.36^a^	2.98 ± 0.13^c^	1.18 ± 0.06^d^
Total *N* (%)	0.14 ± 0.01^b^	0.31 ± 0.03^a^	0.11 ± 0.01^bc^	0.06 ± 0.02^c^
Avail.P (mg kg^−1^)	88.11 ± 13.57^b^	136.41 ± 13.57^a^	50.37 ± 2.89^c^	29.89 ± 1.71^d^
Exch.K (mg kg^−1^)	1618.06 ± 133.92^b^	2166.36 ± 133.92^a^	128.29 ± 7.59^c^	56.81 ± 2.78^d^
Exch.Ca (mg kg^−1^)	8274.76 ± 333.07^b^	9131.06 ± 333.07^a^	2085.09 ± 136.25^c^	1875.33 ± 168.87^c^
Exch.Mg (mg kg^−1^)	1255.81 ± 93.65^b^	2044.92 ± 93.65^a^	638.43 ± 54.77^c^	564.73 ± 53.85^c^

^a‐d^ Denotes significant differences (*p ≤* 0.05) determined by one‐way ANOVA followed by Tukey's HSD post hoc test.

### 3.2 Diversity, Taxonomic Distribution, and Community Composition

**FIGURE 3:**
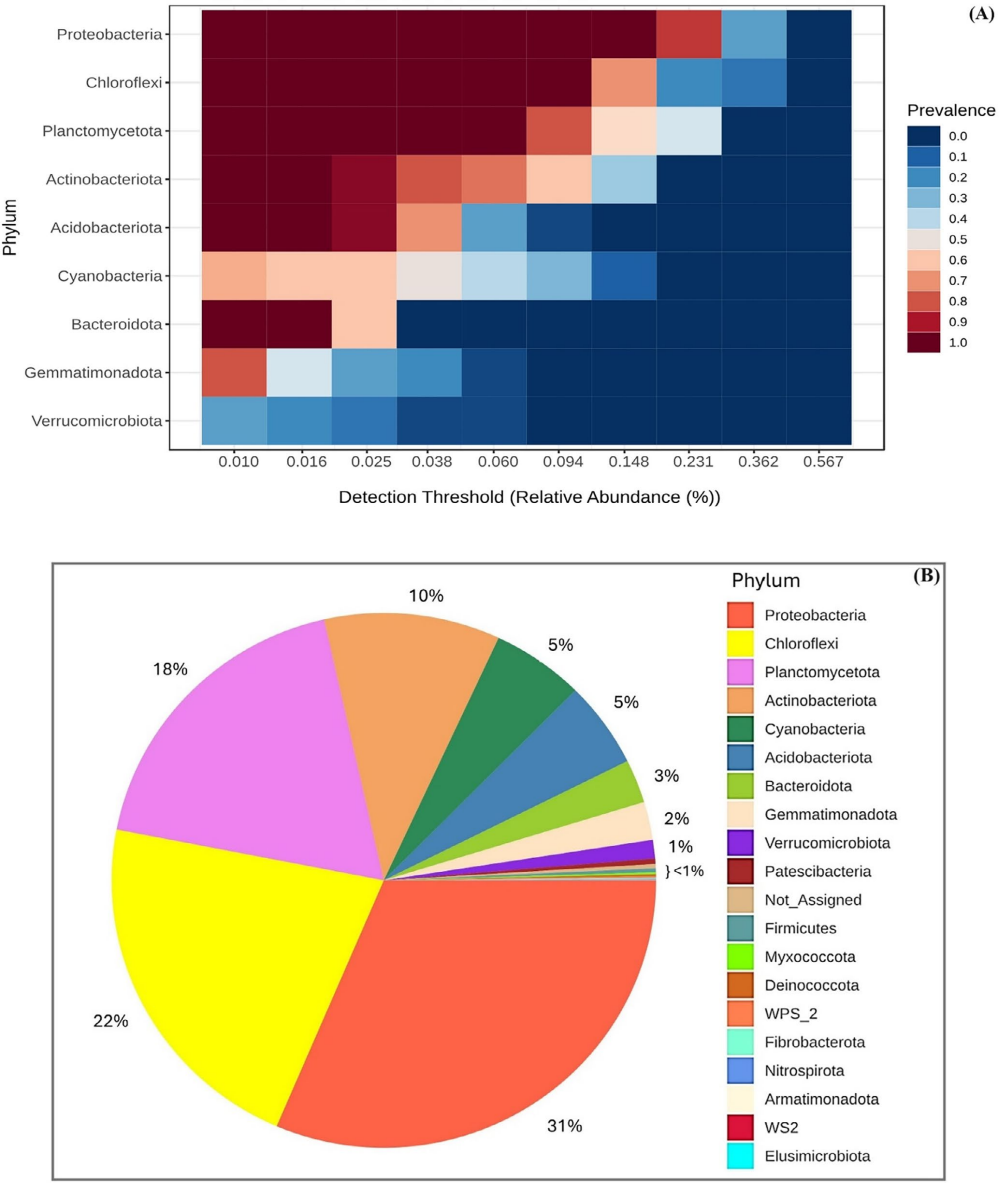
Microbial community composition at the phylum level in soil samples. (A) Heatmap analysis of core microbiome prevalence from low to high relative abundance. The color gradient from blue to red indicates increasing prevalence of each phylum, highlighting core taxa consistently present across soil samples. (B) Pie chart of taxonomic composition demonstrates the relative abundance of microbial phyla within the soil microbiome. This visual represented the taxonomic distribution, with each segment's size corresponding to the proportion of the community attributed to each phylum.

A total of 30 phyla, 69 classes, 160 orders, 196 families, and 340 genera were identified in this study. The core microbiome in soil samples was explored to elucidate the composition and relative abundance of microbial phyla that play pivotal roles in the soil ecosystem. Utilizing a dual‐visual approach, a heatmap and a pie chart were integrated to provide a multidimensional view of microbial prevalence and abundance across all soil samples. The heatmap delineates the prevalence of various microbial phyla at distinct detection thresholds, revealing Proteobacteria as the most predominant phylum, consistently present across varying abundance levels (Figure 3A).

Complementing the heatmap, the pie chart quantifies the composition of the microbial community, with Proteobacteria comprising 31%, followed by Chloroflexi at 22% and Planctomycetota at 18% (Figure 3B). These proportions underscore the dominant roles of these phyla in carbon cycling and OM decomposition. Together, these visualizations not only highlight the preeminence of Proteobacteria but also reveal the extensive diversity within the soil microbiome, illustrating a complex interplay of microbial entities that sustain soil health and ecosystem functionality. This dual‐visualization approach deepens our understanding of the soil microbiome's structure and functional dynamics, offering a comprehensive snapshot of its biodiversity at the phylum level.

**FIGURE 4:**
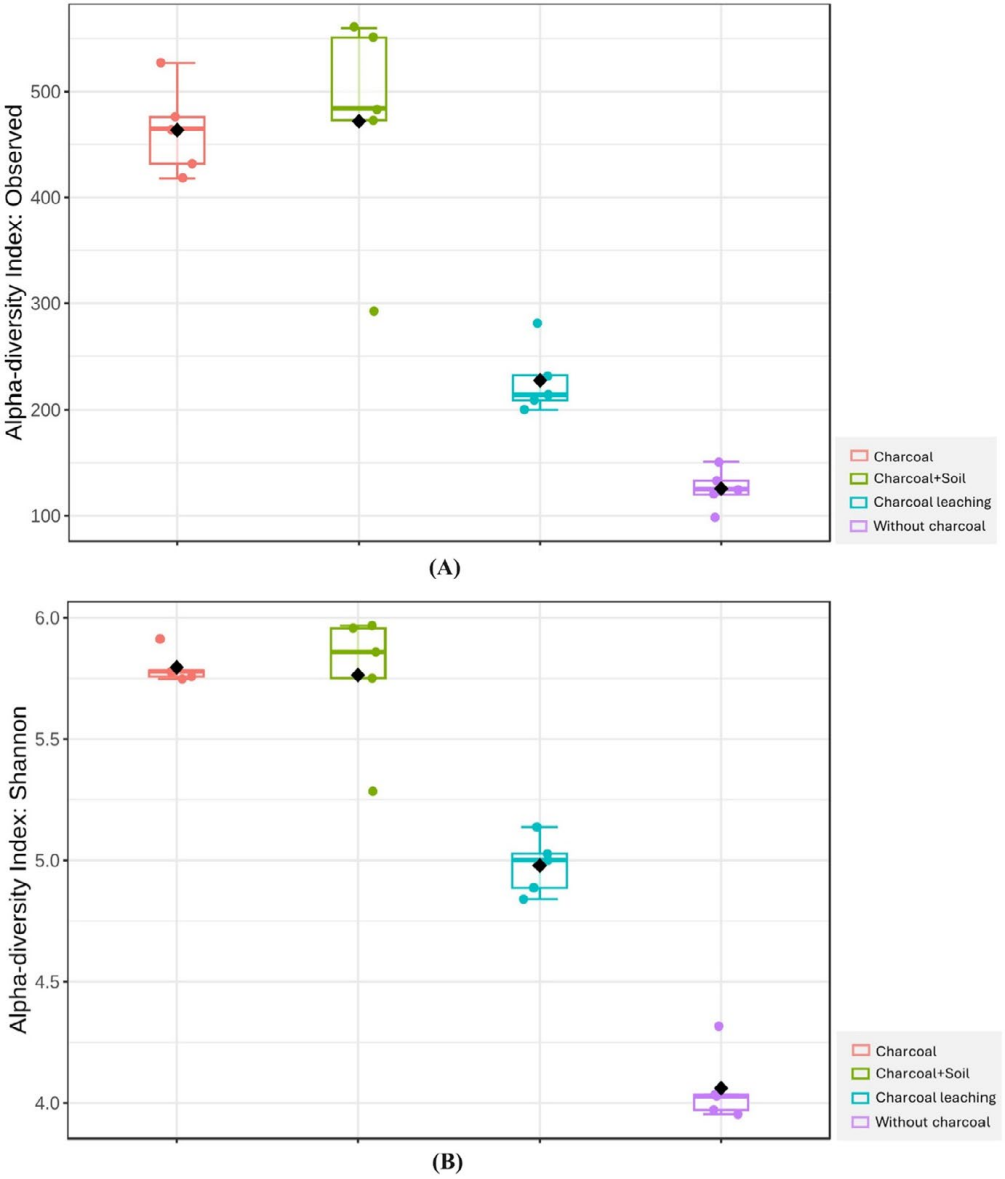
Alpha diversity indexes of soil bacteria under different layers. (A) Observed richness, (B) Shannon index.

The alpha diversity index, incorporating both observed richness (Figure 4A) and the Shannon index (Figure 4B), was utilized to analyze the microbial community's richness and diversity across various soil layers. The analysis identified significant differences (*p* < 0.05) in observed richness and Shannon diversity between all layers, except between the charcoal layer and the charcoal‐mixed soil layer (Figure 4A,B). This suggests that these two layers achieved similarly high levels of microbial diversity, distinguishing them from other layers. The results highlighted the impact of charcoal on enhancing soil microbial diversity, with the charcoal layer exhibiting higher values in observed species richness and Shannon diversity index relative to other layers. The surface layer of charcoal‐mixed soil also demonstrated augmented microbial diversity, albeit slightly less than that of the charcoal layer. In contrast, the lowest observed richness and Shannon diversity were detected in the deeper layer without charcoal.

**FIGURE 5:**
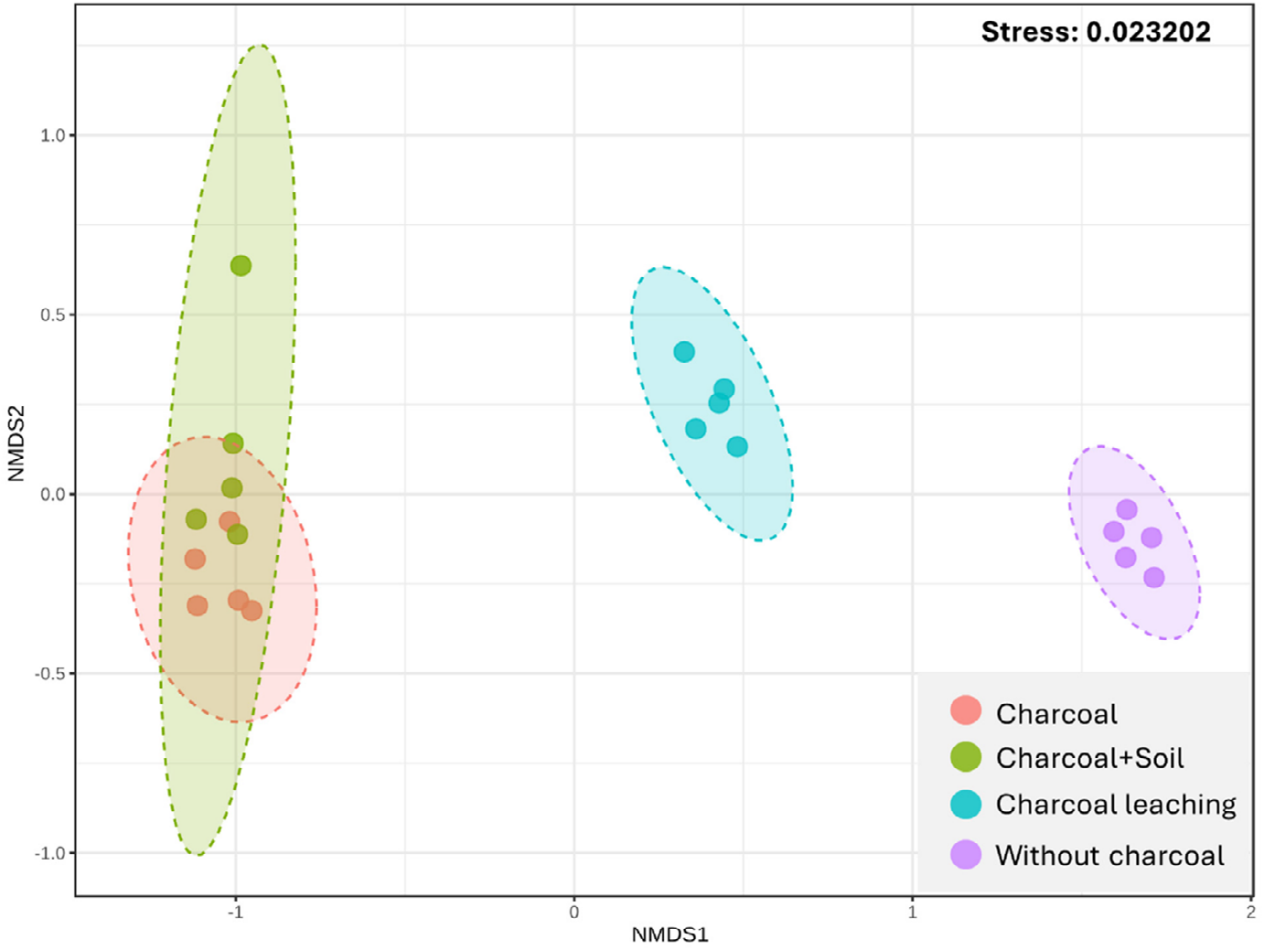
Non‐metric multidimensional scaling (NMDS) analysis of bacterial communities, based on Bray–Curtis dissimilarity, each marker denotes an individual community. The closeness of these points indicates a higher degree of compositional similarity between the communities.

The NMDS analysis demonstrated the separation among groups of four layers, reflecting distinct microbial community structures. Statistical validation through non‐parametric multivariate analysis of variance (NPMANOVA) indicated that the samples treated with charcoal, either in its pure form or mixed with soil, formed a cluster that was not statistically different from each other (*p* = 0.215), suggesting a similar impact on microbial communities by both layers. In contrast, samples subjected to charcoal leaching exhibited statistically significant differences (*p* < 0.05) from both the charcoal and charcoal‐mixed soil groups, highlighting the influence of leaching on altering microbial compositions. Moreover, the soil group without any charcoal was significantly distinct from the charcoal‐containing groups (*p* < 0.05), emphasizing the profound impact of charcoal amendments on enhancing microbial diversity (Figure 5).

**FIGURE 6:**
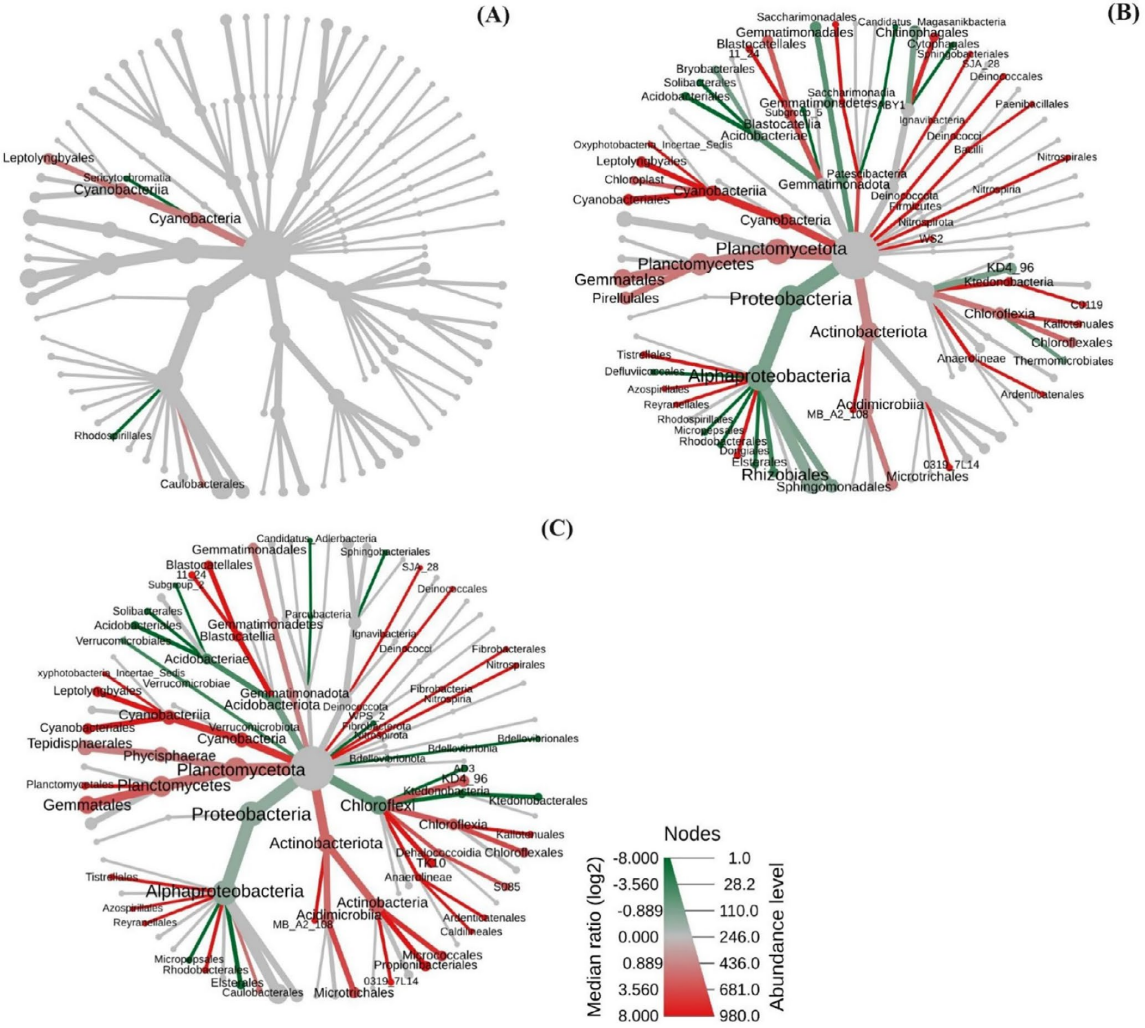
Heat tree visualization of taxonomic differences across layers. (A) Taxonomic differences between charcoal layer and charcoal‐mixed soil (0–2 cm), (B) taxonomic differences between charcoal layer and charcoal leaching layer (2–7 cm), and (C) taxonomic differences between charcoal layer and without charcoal layer (7–15 cm).

The heat tree analysis illustrates shifts in the bacterial community when comparing layers with only charcoal to those mixed with charcoal and soil at a depth of 0–2 cm (Figure 6A). This analysis identifies taxa that have undergone significant changes, which are labeled at the corresponding phylogenetic nodes (Wilcoxon Rank Sum test, *p* < 0.05). A red branch signifies an increase in abundance relative to the charcoal‐only layer, whereas a green branch indicates a decrease. The results revealed that the Cyanobacteria phylum showed a significant increase in abundance in the charcoal‐mixed soil layer.

In comparison with the charcoal leaching layer (Figure 6B), there was a noted reduction in the abundance of Proteobacteria at the phylum level. Within the class Alphaproteobacteria, significant increases were observed in the orders Tistrellales, Azospirillales, Reyranellales, and Dongiales. However, the orders Defluviicoccales, Rhodospirillales, Micropepsales, Rhodobacterales, Elsterales, Rhizobiales, and Sphingomonadales experienced significant decreases. Conversely, within the phylum Planctomycetota, all observed orders, specifically Gemmatales and Pirellulales, demonstrated a significant increase in abundance.

A similar pattern was observed when comparing layers without charcoal (Figure 6C). Proteobacteria also displayed a decline at the phylum level. Notable increases within Alphaproteobacteria were restricted to the orders Tistrellales, Azospirillales, Reyranellales, Rhodobacterales, and Caulobacterales. The orders Micropepsales and Elsterales detected significant decreases. Conversely, within Planctomycetota, the orders Tepidisphaerales, Planctomycetales, and Gemmatales displayed notable increases.

### 3.3 Abundance of Bacterial Genera

**FIGURE 7:**
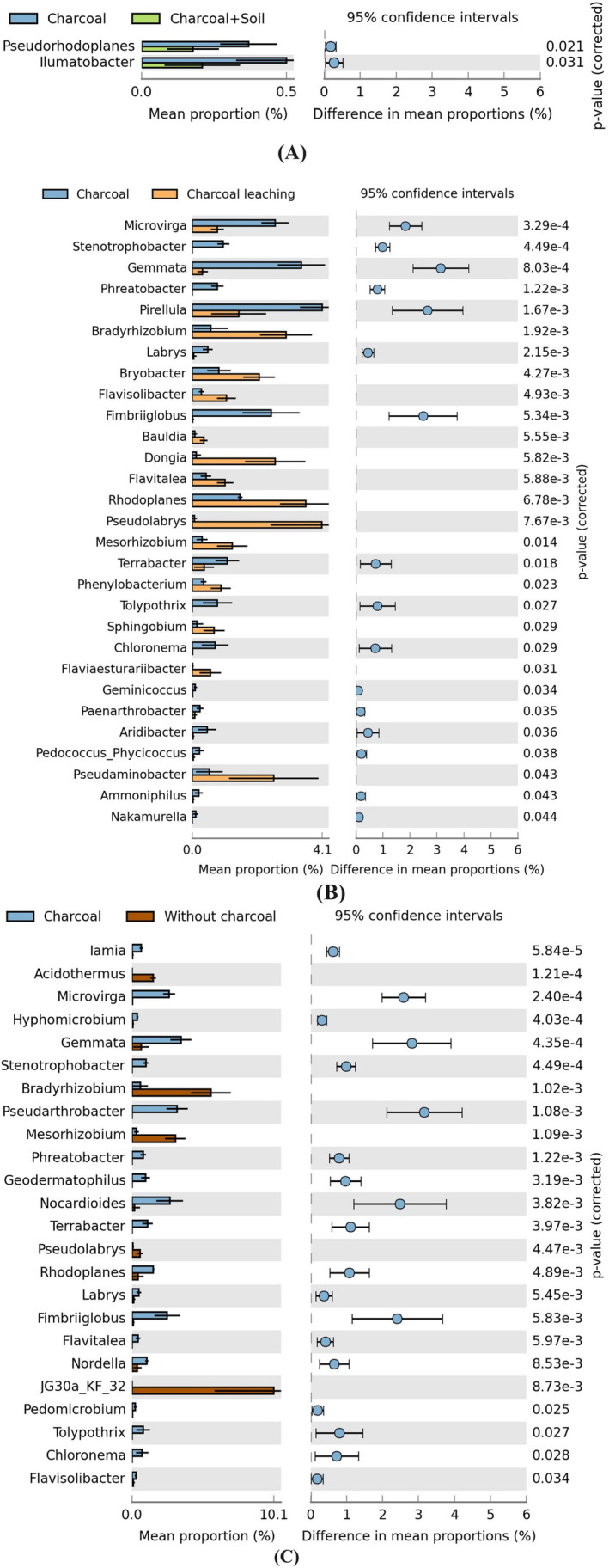
Differential abundance of bacterial genera. (A) Differences in genera between the charcoal layer and charcoal with soil (0–2 cm), (B) differences in genera between the charcoal layer and charcoal leaching layer (2–7 cm), and (C) differences in genera between the charcoal layer and soil without charcoal incorporation (7–15 cm).

Comparative analysis between layers involving charcoal and those combining charcoal with soil revealed that two bacterial genera, *Pseudorhodoplanes* and *Ilumatobacter*, exhibited statistically significant differences in relative abundance (*p* < 0.05) (Figure 7A). Moreover, when comparing charcoal layers to those involving charcoal leaching layers, 29 bacterial genera showed statistically significant differences in their presence (*p* < 0.05). The most abundant genera were *Pseudolabrys*, *Rhodoplanes*, *Bradyrhizobium*, *Dongia*, and *Pseudaminobacter* (Figure 7B). Comparing charcoal layers to soils without charcoal incorporation also identified 24 bacterial genera with statistically significant variations in abundance. The most abundant genera were JG30a‐KF‐32, *Bradyrhizobium*, and *Mesorhizobium* (Figure 7C).

**FIGURE 8:**
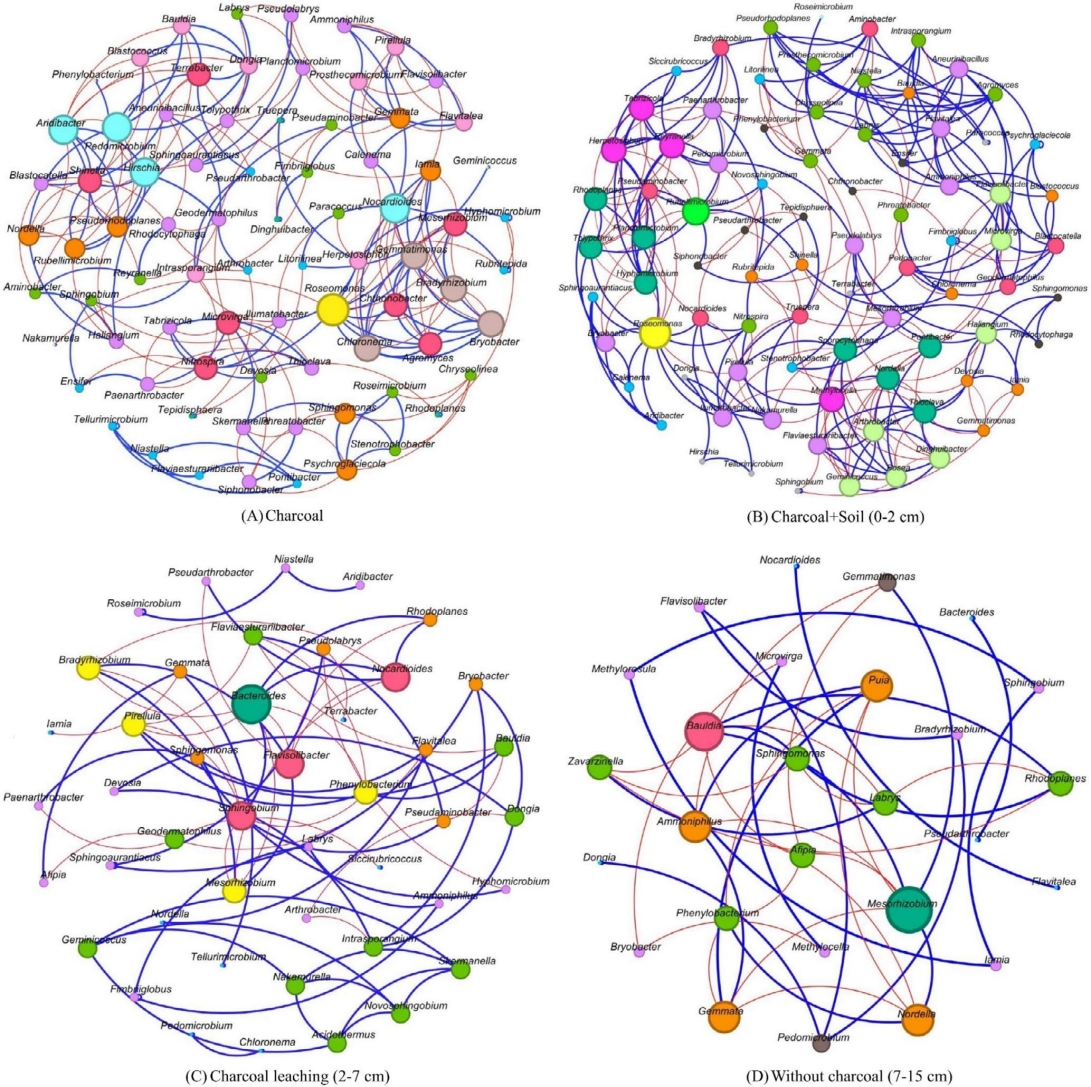
Network analysis of bacterial communities across four layers. Interaction networks for each layer are shown: (A) charcoal layer, (B) charcoal‐mixed soil layer, (C) charcoal leaching layer, and (D) without charcoal layer.

In the charcoal‐only layer, the network comprised 78 nodes, each representing different bacterial identities, with a total of 288 interactions among the community members. *Rosemonas*, *Nocardioides*, *Hirschia*, *Pedomicrobium*, and *Aridibacter* exhibited the most interactions with other genera (Figure 8A). In the charcoal‐mixed soil layer, the network comprised 83 nodes, each corresponding to different bacterial identities, with a total of 352 interactions. *Rosemonas*, *Rubellimicrobium*, *Herpetosiphon*, *Reyranella*, and *Tabrizicola* exhibited the most complex interactions (Figure 8B). Moreover, *Bacteroides* exhibited the most complex interactions in the charcoal leaching layer, which displayed a smaller network with 45 nodes, each representing different bacterial identities, and a total of 115 interactions (Figure 8C). However, the most reduced network was detected in soil without any charcoal layer, consisting of 27 nodes, each representing unique bacterial identities, and only 29 interactions. This indicates the lowest complexity and connectivity of bacterial communities compared to the other layers. *Mesorhizobium* exhibited the most complex interactions in this layer (Figure 8D).

## 4 Discussion

### 4.1 Effect of Charcoal on Soil Physicochemical Properties

The charcoal layer, as expected, retains most of the nutrients (TN content, avail.P, exch.K, exch.Ca, and exch.Mg), gradually releasing them into the soil surface. The available nutrients can then be transported to deeper layers by water percolation (Pu et al. 2019). Jobbágy and Jackson (2001) also revealed that the increasing concentration of nutrients with depth is related to leaching from the topsoil. At high fire intensity, nitrogen is released into the atmosphere as gas forms during combustion (Kalidas‐Singh et al. 2021) and generally takes a longer time to recover (Arunrat et al. 2023c).

Charcoal contains both macropores and micropores, which have a high potential for adsorption on their surface area (Keech, Carcaillet, and Nilsson 2005). Within these pores, nutrients such as N, avail. P, Ca, Mg, and K are adsorbed after biomass combustion and are later released into the soil (Ulery, Graham, and Bowen 1993; Glaser et al. 2002). This is consistent with the findings of the present study, which show that soil nutrient contents (SOM, TN, avail.P, exch.K, exch.Ca, and exch.Mg) in the charcoal‐mixed soil layer were significantly higher compared to those in the other layers (Table 2). The increase in soil pH after the fire is likely due to the oxidation of organic material, which releases alkaline cations (Ca, Mg, K, Na) along with charcoal and ash (Certini 2005; Glaser et al. 2002). Moreover, an increase in pH can reduce the amount of exchangeable toxic metals, such as aluminum (Al) (Steiner et al. 2007). As charcoal is a form of stable carbon, it contributes to the increased SOM when fire converts plant material into char and ash, which then become incorporated into the soil (González‐Pérez et al. 2004). Chungu et al. (2020) also found the SOM on burned sites in *Eucalyptus grandis* plantations in Zambia increased by 32% and 72% 1‐ and 3‐years after fire, respectively. The TN content, avail.P, exch.K, exch.Ca, and exch.Mg tend to increase after a fire due to the release from dead roots and N‐containing organic compounds during burning (Rivas et al. 2012) and the addition of pyrolyzed materials after fire (Grogan, Burns, and Chapin 2000).

### 4.2 Effect of Charcoal on Soil Bacterial Abundances and Community Compositions

Fire alters soil properties and changes microhabitats, thereby impacting the microbial community composition (Hamman, Burke, and Stromberger 2007; Pereira et al. 2019) and function by changing the taxa and the functional genes (D'Ascoli et al. 2005; Prendergast‐Miller et al. 2017). After 1 year of fire in RSC, Proteobacteria was found to be the most dominant phylum throughout the soil profile (Figure 3A,B). Moreover, the charcoal‐only layer on the surface and the charcoal‐mixed soil layer exhibited significantly higher richness and diversity of soil bacteria, while the lowest observed richness and Shannon diversity were detected in the deeper layer without charcoal (Figure 4A,B). Prendergast‐Miller et al. (2017) detected an increase in the abundance of Proteobacteria, Actinobacteria, and Firmicutes in burnt sites. Similarly, Rodríguez et al. (2017) found that microbial biomass and diversity were higher in burnt soils compared to control soils in Sierra de Aznalcóllar, Southern Spain. This can be explained by two reasons. First, the fire was not severe enough to eliminate the soil bacteria, which were heat‐resistant (Pulido‐Chavez et al. 2023). Kalidas‐Singh et al. (2021) reported that soil temperatures during burning can reach between 350°C and 700°C at a depth of 1 cm, sterilizing the top layer. However, these temperatures decrease rapidly with depth, creating favorable conditions for early‐succession microorganisms to dominate in the subsoil with minimal competition. Second, the nutrient‐rich and reduced soil acidity from charcoal and ash facilitated the rapid growth of soil bacteria, particularly Proteobacteria (Glaser et al. 2002; Kolb, Fermanich, and Dornbush 2009). In addition, the porous structure and large surface area of charcoal provide a habitat for beneficial soil microorganisms (Pietikäinen, Kiikkilä, and Fritze 2000).

The Cyanobacteria showed a significant increase in abundance in the charcoal‐mixed soil layer (Figure 6A). Due to the nutrient richness of this layer (Table 2), it promotes the early recolonization by cyanobacteria (Büdel et al. 2016). This is consistent with Weber et al. (2016), who revealed that Cyanobacteria are considered early colonizers after a fire. The abundance of Proteobacteria declined with increased depth, as expected (Figure 6B,C), due to a decrease in soil nutrient content, which is consistent with the findings of Li et al. (2022). Meanwhile, Planctomycetota demonstrated a significant increase in abundance in both the charcoal‐leached soil and the soil without charcoal (Figure 6B,C). Kaboré, Godreuil, and Drancourt (2020) found that Planctomycetes are slow‐growing bacteria that thrive in nutrient‐poor oligotrophic environments, characteristics that may contribute to their prevalence in areas containing charcoal leaching.

Charcoal and charcoal‐soil containing revealed that two bacterial genera, *Pseudorhodoplanes* and *Ilumatobacter*, exhibited statistically significant differences in relative abundance (*p* < 0.05) (Figure 7A). This may be due to an increase in charcoal and ash, which changes the soil pH and macronutrient levels. Xu et al. (2018) reported that *Pseudorhodoplanes* plays a key role in hydrocarbon degradation. Tirandaz et al. (2015) found that *Pseudorhodoplanes sinuspersici* has optimal activity at a temperature of 30°C and a pH of 7 while demonstrating tolerance within a pH range of 5.5–8 and a temperature range of 15°C–35°C. Moreover, members of the genus *Ilumatobacter* can grow in alkaline conditions (pH 6–10) (Matsumoto et al. 2013; Asem et al. 2018) and are known to produce the enzyme esterase lipase (Matsumoto et al. 2013).

In charcoal‐leached soil, the most abundant genera were *Pseudolabrys*, *Rhodoplanes*, *Bradyrhizobium*, *Dongia*, and *Pseudaminobacter* (Figure 7B). The available nutrients in this layer were leached from the top layer, and competition among soil bacteria may be high. Thus, the abundant genera in this layer need to have specific adaptations to survive. Eo and Park (2016) found that *Pseudolabrys* increased in fertilization treatments with low P levels. *Rhodoplanes* are involved in the N cycle, including N fixation, nitrate reduction, and denitrification (Cui et al. 2017). *Rhodoplanes* could increase N availability in the soil via N fixation while simultaneously decreasing available N through denitrification (Zhang et al. 2022). Moreover, *Bradyrhizobium* is among the genera of N‐fixing bacteria (Yang et al. 2021). Halder et al. (1990) demonstrated that *Bradyrhizobium* can promote the dissolution of rock phosphate. Guo et al. (2024) reported that *Dongia* may play an important role in the degradation of polycyclic aromatic hydrocarbons (PAHs) in biochar‐treated rhizosphere soil. Some species within the genus *Pseudaminobacter* have shown the ability to degrade environmental pollutants such as the N‐methylcarbamate insecticide prothiocarbamate (Kim et al. 2017) and methyl parathion (Zhang et al. 2005).

In soil layers not containing charcoal, the most abundant genera were JG30a‐KF‐32, *Bradyrhizobium*, and *Mesorhizobium* (Figure 7C). Due to the low nutrient levels in this layer compared to the other layers (Table 2), the abundant soil bacteria have developed the proficiency to adapt for survival. Previous studies (Uroz et al. 2010; VanInsberghe et al. 2015; Jones et al. 2016) have reported that *Bradyrhizobium* is a slow‐growing soil bacterium. Forming N_2_‐fixing symbioses is also an ability of *Bradyrhizobium* (Jones et al. 2016) and *Mesorhizobium* (Menéndez et al. 2020; Colombi et al. 2023), which helps to increase N availability for their activities and plants.

Soil bacteria with high interactions with other genera indicate the symbiosis of soil bacterial communities, which affects the composition, diversity, and dynamics of microbial populations (Ryan and Dow 2008; Schlatter et al. 2015). As shown in Figure 8A–D, *Rosemonas*, *Rubellimicrobium, Bacteroides*, and *Mesorhizobium* exhibited the most interactions with other genera in charcoal layer, charcoal‐containing soil, charcoal‐leached soil, and without charcoal layer, respectively. *Roseomonas* is commonly found in soil (Dong et al. 2014; Kim and Ka 2014). Rat et al. (2021) revealed that *Roseomonas* can uptake extracellular nitrate and nitrite. *Rubellimicrobium* is reported as a denitrifying bacterium that serves as an aerobic denitrifier (Fan et al. 2022). *Bacteroides* has the potential to provide energy for adjacent bacteria (Zafar and Saier 2021). It is an obligate anaerobe that can only grow and thrive in environments without oxygen (Rocha et al. 2003; Baughn and Malamy 2004). Moreover, *Mesorhizobium* is widely known as an N‐fixing bacterium associated with legumes. *Mesorhizobium* can promote plant growth and is recommended as an alternative for non‐legume crops to avoid using chemical fertilizers (Menéndez et al. 2020). The interaction of these bacteria with other genera in each soil layer represents steady coexistence under certain nutrient levels, whereas some genera may be outcompeted under nutrient‐restricted conditions (Hibbing et al. 2010). A higher complexity of soil bacteria interactions indicates high bacterial diversity due to the presence of highly competitive bacteria, as shown in the charcoal layer (Figure 8A) and the charcoal‐mixed soil layer (Figure 8B). This is consistent with the study by Domin et al. (2018), which found that competitive bacteria resulted in increased alpha diversity.

## 5 Conclusions

This study demonstrates that fire‐deposited charcoal significantly alters soil physiochemical properties and enhances microbial diversity in RSC in Northern Thailand. Charcoal led to higher pH and electrical conductivity in the charcoal layer, with notable differences in soil texture across layers, including the highest sand and silt content in the charcoal‐mixed soil layer (0–2 cm). Soil OM and total nitrogen were significantly higher in the charcoal‐mixed layer compared to deeper layers, indicating improved nutrient retention due to charcoal presence. Enhanced microbial diversity was observed in the charcoal and charcoal‐mixed soil layers, with Proteobacteria, Chloroflexi, and Planctomycetota dominating across all soil samples. The bacterial genus *Ilumatobacter* exhibited significant changes in abundance in response to the presence of charcoal. *Pseudolabrys* was more abundant in charcoal‐leached soil, whereas JG30a‐KF‐32 showed higher abundance in soil without charcoal. Shifts in Proteobacteria and Planctomycetota abundance were evident in the charcoal leaching and non‐charcoal layers. Network analysis indicated more complex bacterial interactions in the charcoal‐mixed soil layer, with reduced network complexity observed in the charcoal leaching layer and the layer without charcoal. This suggests lower connectivity and interaction among bacterial communities in these layers. These findings imply that charcoal provides a favorable environment for diverse and interactive bacterial communities, potentially benefiting soil health and fertility. While these results underscore the potential of charcoal amendments in enhancing soil properties and microbial diversity, further research is needed to confirm these findings and explore their broader implications for soil health and agricultural productivity.

## Author Contributions

**Noppol Arunrat:** conceptualization (lead), data curation (lead), formal analysis (equal), funding acquisition (lead), investigation (lead), methodology (equal), writing – original draft (lead), writing – review and editing (lead). **Toungporn Uttarotai:** formal analysis (equal), methodology (equal), writing – original draft (equal). **Praeploy Kongsurakan:** methodology (equal), writing – original draft (equal). **Sukanya Sereenonchai:** conceptualization (equal), investigation (equal), writing – original draft (equal). **Ryusuke Hatano:** methodology (equal), supervision (lead), writing – original draft (equal).

## Conflicts of Interest

The authors declare no conflicts of interest.

## Data Availability

The sequencing data relevant to this research are available at the National Center for Biotechnology Information (NCBI), recorded under the BioProject accession number PRJNA1085479.
